# Reproducibility of different screening classifications in ultrasonography of the newborn hip

**DOI:** 10.1186/1471-2431-10-98

**Published:** 2010-12-24

**Authors:** Christian D Peterlein, Karl F Schüttler, Stefan Lakemeier, Nina Timmesfeld, Christian Görg, Susanne Fuchs-Winkelmann, Markus D Schofer

**Affiliations:** 1 Department of Orthopaedics and Rheumatology, University Hospital Giessen and Marburg, Marburg, Germany; 2 Institute of Medical Biometry and Epidemiology, Philipps University Marburg, Germany; 3 Department of Internal Medicine, Ultrasound-Laboratory, University Hospital Giessen and Marburg, Marburg, Germany

## Abstract

**Background:**

Ultrasonography of the hip has gained wide acceptance as a primary method for diagnosis, screening and treatment monitoring of developmental hip dysplasia in infants. The aim of the study was to examine the degree of concordance of two objective classifications of hip morphology and subjective parameters by three investigators with different levels of experience.

**Methods:**

In 207 consecutive newborns (101 boys; 106 girls) the following parameters were assessed: bony roof angle (α-angle) and cartilage roof angle (β-angle) according to Graf's basic standard method, "femoral head coverage" (FHC) as described by Terjesen, shape of the bony roof and position of the cartilaginous roof. Both hips were measured twice by each investigator with a 7.5 MHz linear transducer (SONOLINE G60S^® ^ultrasound system, SIEMENS, Erlangen, Germany).

**Results:**

Mean kappa-coefficients for the subjective parameters shape of the bony roof (0.97) and position of the cartilaginous roof (1.0) demonstrated high intra-observer reproducibility. Best results were achieved for α-angle, followed by β-angle and finally FHC. With respect to limits of agreement, inter-observer reproducibility was calculated less precisely.

**Conclusions:**

Higher measurement differences were evaluated more in objective scorings. Those variations were observed by every investigator irrespective of level of experience.

## Background

Since its introduction in 1980, ultrasonography (US) of the newborn hip has gained widespread acceptance in the screening and diagnosis of developmental hip dysplasia (DDH) [1-5]. Over time, various screening methods and classifications were developed. The most widely used method of evaluating ultrasonograms in newborns is the measurement of the bony roof angle (α-angle) and the cartilage roof angle (β-angle) according to Graf [6-8]. However, some investigators demonstrated that these methods were susceptible to measurement errors, particularly in newborns [9,10]. A technique based on the measurement of distances was later developed by Terjesen [11,12] and Morin [13].

Discrepancy in measurement may be due to the variability in the US examination itself and in its interpretation. Studies demonstrated that both the performance of US and its interpretation influence the results and potential treatment [10,14-16]. The aim of our study was to analyze the reproducibility of two objective classifications and descriptive parameters in newborn hip US and the influence of investigators' level of experience. Unlike in other studies, all three investigators both performed the US and provided the interpretation of their own images in a blinded fashion.

## Methods

The hips of 207 consecutive newborns (101 boys, 106 girls) were prospectively screened. The study was conducted in accordance with the Declaration of Helsinki and approved by the ethics committee of the University of Marburg, Germany. Informed consent was obtained from both parents. US was performed on each newborn by three investigators with different levels of experience - an experienced paediatric orthopaedic surgeon (CP), a senior orthopaedic surgeon (MS), and a trained medical student (KS). The former two investigators attended several formal US training courses. The medical student attended basic US training and theoretical lessons on Graf's and Terjesen's techniques. We used a mobile SONOLINE G60S^® ^ultrasound system (SIEMENS, Erlangen, Germany), equipped with a 7.5 MHz linear array probe. According to Graf, newborns up to week 4 of life should be examined with a linear transducer with a minimum frequency of 7.5 MHz, for precise measurement of small anatomical structures [17]. The software of the SONOLINE G60S^® ^produces a standard projection of the image, which can be viewed and interpreted in the anterior-posterior view, as if on a plain radiograph. Adjustments in processing had been previously carried out by the Head of the Ultrasound Laboratory (CG).

**Figure 1:**
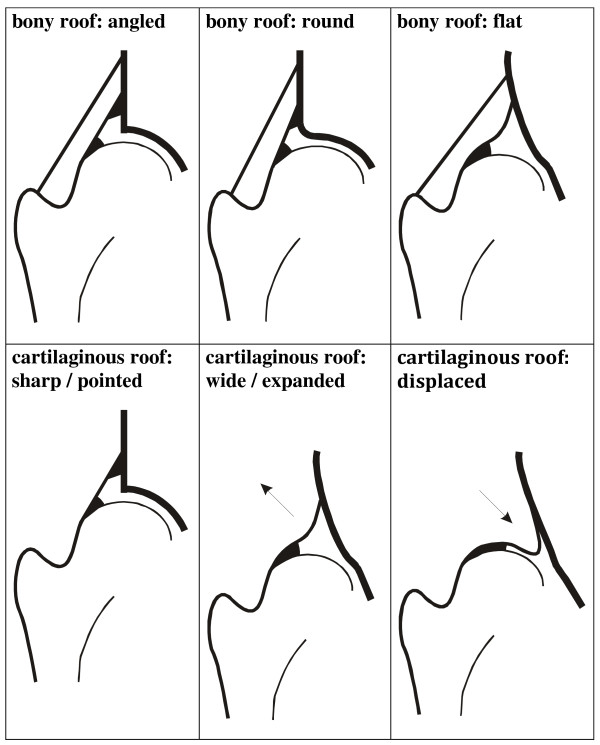
**Characterization of descriptive parameters**.

**Figure 2:**
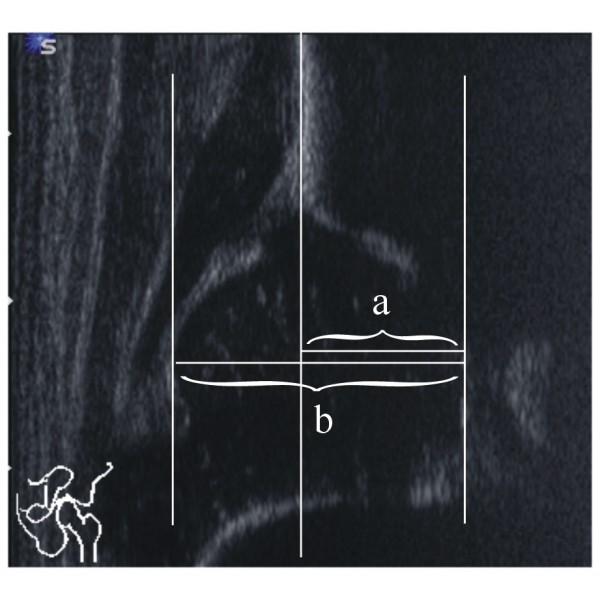
**Measurement of the femoral head coverage (FHC)**.

**Figure 3:**
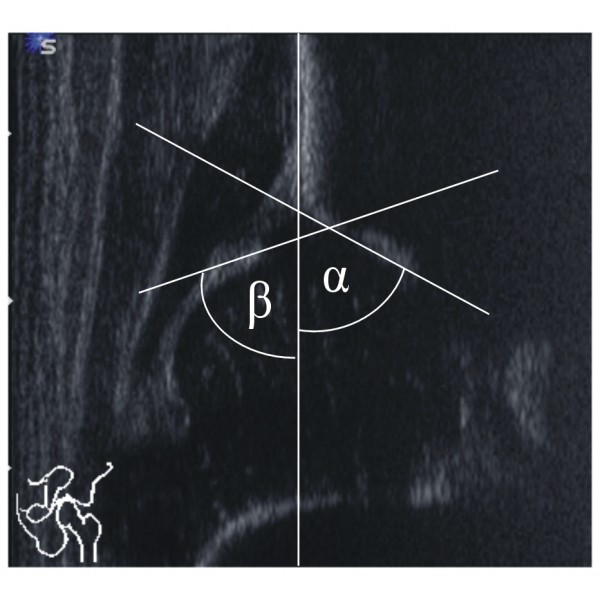
**Measurement of the α- and β-angle**.

Both hips were measured twice by each investigator. The examination was conducted in an infant bassinet, which allowed for standardized positioning and scanning. According to Graf, standard images through the deepest part of the acetabulum were obtained in the coronal plane. The three landmarks were considered: the lower limb of the os ilium, the mid portion of the acetabular roof, and the labrum. The pictures were stored on the SONOLINE G60S^® ^hard drive, and then printed on high-quality paper strips (thermal paper K65HM-CE, Mitsubishi, Japan) by a statistician (NT) who was not involved in the examinations. The strips were randomized to generate blinded conditions. Each investigator independently evaluated his own hard-copy strips 4 weeks later. Measurements were performed manually. In a standardized manner, two descriptive parameters - the shape of the bony roof and the position of the cartilaginous roof - were assigned first (Figure 1). After drawing a reference line, two parallel lines, (a) from the acetabular floor to the reference line, and (b) from the same point on the acetabular fossa to the most lateral part of the cartilaginous femoral head were marked (Figure 2). The distances were measured in millimeters, and femoral head coverage according to Terjesen [11,12] was calculated by the formula a/b × 100%. Finally, the bony roof angle (α-angle) and the cartilage roof angle (β-angle) were measured [7,8] (Figure 3). Thus, each investigator examined a total of 414 hips (828 hard copy strips). Examiners did not observe each other nor did they communicate about their interpretations until the end of the study.

### Statistical analysis

The mean of the 6 observations from each hip was computed for α- and β-angle and femoral head coverage (FHC) and hips were thus classified. As in previous studies [18,19] hip types were combined to form 4 main groups: type I = normal; type IIa = immature; type IIc/D = minor dysplasia; and types III/IV = major dysplasia. For the continuous outcomes, α- and β-angle and femoral head coverage (FHC), intra-observer agreement was obtained by the mean difference between two series of measurements and related limits of agreement [20]. Inter-observer agreement between two observers was measured by mean difference and general limits of agreement [21].

For nominal outcomes, such as shape of the bony roof and position of the cartilaginous roof, Cohen's kappa coefficient and the percentage of agreement were computed for both intra- and inter-observer agreement. For inter-observer agreement between two observers, the mean of Cohen's kappas, obtained from the four pairs of measurements, was calculated. Inter-observer agreement between all three observers was measured by the mean of Light's kappas, obtained from the nine combinations. Similarly, the percentages of agreement were calculated. All computations were done by statistical software R [22].

## Results

207 consecutive newborns (101 male, 106 female) were screened, at an average age of 2.64 days of life (range 1 - 8 days). A total of 2484 hard copy strips were evaluated. The mean α-angle was 64.9° (± 3.7°; range 46.3° - 75.2°), the mean β-angle was 61.4° (± 4.8°; range 50.5° - 91.3°), and the mean femoral head cover value (FHC) was 61.4% (± 5.0%; range 49.4% - 90.8%). In the male study population the mean α-angle was 65.9° (± 3.3°; range 55.0° - 75.2°), the mean β-angle was 60.3° (± 4.1°; range 50.5° - 74.2°), and the FHC was 60.3% (± 4.4%; range 49.4% - 74.4%). The female study population demonstrated an average α-angle of 63.9° (± 3.8°; range 46.3° - 72.8°), β-angle of 62.4° (± 5.2°; range 51.7° - 91.3°), and FHC value of 62.5% (± 5.2%; range 51.6% - 90.8%). Both the α-angle and the FHC demonstrated a significant difference between sexes (p < 10^-7 ^and p < 10^-5^). There was no statistically significant difference between the left and the right hips. Terjesen defined hips with femoral head cover <47% (male) and <44% (female) as pathological. These values were not measured in our cohort. According to Graf's classification, 31 hips (7.5%) were immature (n = 31) and one hip (0.2%) dysplastic (Additional file 1).

### Objective scorings

The best results with respect to limits of agreement were achieved for the α-angle (mean range: -5.12 - +5.61), followed by the β-angle (mean range: -10.12 - +10.09), and finally for FHC (mean range: -10.52 - +11.03). The experienced pediatric orthopaedic surgeon achieved the most accurate reproducibility of the Graf classification. The Terjesen classification was reproduced most accurately by the medical student (Additional file 2). For all parameters, the inter-observer reproducibility was calculated as less precise; those variations were observed in all three investigators, irrespective of level of experience. The kappa statistics indicated moderate agreement.

### Descriptive scorings

The mean kappa-coefficients for the subjective parameters, shape of the bony roof (0.97) and position of the cartilaginous roof (1.0), demonstrated high intra-observer reproducibility (Additional file 3). For all parameters, the inter-observer reproducibility was calculated as less precise.

## Discussion

This study was conducted to compare the reproducibility of the Graf and Terjesen methods and to analyze the value of descriptive parameters in newborn hip US. Sonographic measurements of anatomical specimens in a water bath demonstrated comparable reproducibility for the two methods [23] but only a few clinical studies have been published to date [24-26]. Czubak [25] and Falliner [24] found a significant correlation (p < 0.01) between the α-angle and the FHC. Unlike in our study, the β-angle was not measured and the authors calculated contradictory results. Falliner scored 4.1% of the hips as dysplastic according to Terjesen, and 1.2% according to Graf; Czubak found 29% of 657 hips to be "immature" according to Graf, and 14% "suspected dysplastic" according to Terjesen. The definition of pathological hips in measurement techniques, based on the calculation of distances, is inconsistent [11-13]. Assuming that hips with FHC <47% (male) and <44% (female) are pathological, no one in our cohort was affected. Our results, with respect to the Graf (7.5% immature and 0.2% dysplastic) better match the reported frequency of hip dysplasia in Europe [27-29].

The correlation coefficients and the limits of agreement for the measured bony roof angle (α-angle) in our study closely correlate with those found by Roovers [18] and Simon [19]. Dias [30], Bar-On [14], and Ömeroglu [31] published better results for the kappa coefficients. However, unlike in our study, hips were classified as simply "normal" and "abnormal." Since the kappa coefficients depend on true prevalences, studies can only be correctly compared if there is agreement among the group categories.

Further studies demonstrated that examiners tend to report higher variations when determining β-angle compared with α-angle [15,16,32]. This variance is also observed when the angles are measured by the same investigator. In our study, we found no large systematic differences in α-angle and β-angle measurements between the three observers. The relatively high variability of the measured β-angles in our study supports the findings of others [10,14,15,32].

Simon evaluated inter-observer agreement of the Graf classification between a radiology team, orthopaedists, registrars and paediatricians. The four groups were not present when the images were obtained and blinded with respect to anamnesis and clinical examination of the infants. Greatest agreement existed between the paediatricians and the orthopaedists. The authors explained this result by the long-term-experience in these physicians in US.

Unlike previously described studies, the three investigators in this study both performed US on the newborns and analyzed their own results in a blinded fashion. We found no statistically significant difference between investigators' measurements. This was unexpected, since the paediatric orthopaedic surgeon (CP) conducts more than 1000 hip US examinations per year and the medical student (KS), none.

For the parameters shape of the bony roof and position of the cartilaginous roof, kappa statistics indicate excellent intra- and inter-observer agreement. This might be explained by the fact that all investigators, irrespective of their level of experience in clinics, were trained in checking the "principles of the standard plane" accurately - lower limb of the bony ileum in the depth of the acetabular fossa, mid portion of the acetabular roof, and acetabular labrum. However, standardized anatomical identification in US is mandatory. According to Graf, this includes determination of the chondroosseous junction (epiphyseal plate of the femur), femoral head, synovial fold, and joint capsule.

The correct order of the anatomical identification of the newborn hip US is taught in training courses. Hell recently assessed inter- and intra-observer reliability and learning curves in participants after basic, advanced, and final courses in hip US using the Graf method. Improvements in reproducibility gradually occurred in course participants. Measurement discrepancies were seen, particularly in abnormal and poor quality US examinations, and in the measurement of the β-angle [32,33].

There were several limitations to our study. Only one dysplastic hip was found in the study group. Thus, the data lacks reliability for abnormal hips and requires a larger sample size. Moreover, the rapid measurement schedule is prone to induce errors due to resistive newborns, malposition, or tilting of the probe.

## Conclusions

US is a sensitive diagnostic tool in detection and management of DDH. Our study demonstrates that, irrespective of investigator experience, an adequate degree of inter- and intra-observer reliability can be obtained for both objective and descriptive parameters. A standardized method of anatomical identification of landmarks is mandatory.

## Competing interests

The authors declare that they have no competing interests.

## Authors' contributions

Ultrasonography was performed by CDP, KFS and MDS. The initial draft was written by CDP. CG, SL and SFW contributed equally to this work: they advised in the developing of the study protocol and critically revised the manuscript. NT performed data collection, analysis and statistics. All authors participated in the reviewing process and approved the final manuscript.

## Pre-publication history

The pre-publication history for this paper can be accessed here:

http://www.biomedcentral.com/1471-2431/10/98/prepub

## Supplementary Material

Additional file 1 **Distribution of 414 US examinations (mean of 6 observations from each hip), according to Graf**. Click here for file

Additional file 2 **Intra- and inter-observer results of objective parameters (mean difference and limits of agreement, in parentheses)**. Click here for file

Additional file 3 **Intra- and inter-observer results of subjective parameters (mean difference and limits of agreement, in parentheses)**. Click here for file
