# Supplementary material for: Reproducibility of different screening classifications in ultrasonography of the newborn hip
Source: BMC Pediatr. 2010 Dec 24;10:98. doi: 10.1186/1471-2431-10-98 (PMC3022795; doi:10.1186/1471-2431-10-98)
Supplement: Additional file 1 — Distribution of 414 US examinations (mean of 6 observations from each hip), according to Graf. [file 1471-2431-10-98-S1.DOC]

| **Hip Type (Graf Classication)** | **Total; %** |
| --- | --- |
| Mature  (Type Ia/ Ib) | 382; 92.3% |
| Immature  (Type IIa) | 31; 7.5% |
| Minor dysplasia  (Type IIc/D) | 1; 0.2% |
| Major dysplasia  (Type III/IV) | 0; 0% |
