# Supplementary material for: Reproducibility of different screening classifications in ultrasonography of the newborn hip
Source: BMC Pediatr. 2010 Dec 24;10:98. doi: 10.1186/1471-2431-10-98 (PMC3022795; doi:10.1186/1471-2431-10-98)
Supplement: Additional file 2 — Intra- and inter-observer results of objective parameters (mean difference and limits of agreement, in parentheses). [file 1471-2431-10-98-S2.DOC]

| **Intra-observer** |  |  |  |
| --- | --- | --- | --- |
| **Investigator** | **CP** | **MS** | **KS** |
| **α-angle** | 0.25 ( -5.12 - +5.61 ) | -0.68 ( -6.44 - +5.09 ) | 0.37 ( -6.05 - 6.79 ) |
| **β-angle** | -0.38 ( -8.87 - +8.11 ) | 0.57 ( -8.95 - +10.09 ) | 0.14 ( -9.25 - 9.52 ) |
| **FHC** | 0.1 ( -10.52 - +10.72 ) | 0.22 ( -11.06 - +11.5 ) | 0.15 ( -9.39 - 9.69 ) |
| **Inter-observer** |  |  |  |
| **Investigator** | **CP – MS** | **CP – KS** | **MS - KS** |
| **α-angle** | -2.54 (-13.41 – +8.34) | -3.88 (-14.9 – +7.14) | -1.34 (-13.8 – +11.12) |
| **β-angle** | 1.39 (-15.74 – +18.51) | -3.05 (-18.52 – +12.42) | -4.43 (-22.99 – +14.12) |
| **FHC** | 1.74 (-15.68 – +19.16) | 0.11 (-15.52 - +15.75) | -1.63 (-18.44 – +15.18) |
