# Supplementary material for: Reproducibility of different screening classifications in ultrasonography of the newborn hip
Source: BMC Pediatr. 2010 Dec 24;10:98. doi: 10.1186/1471-2431-10-98 (PMC3022795; doi:10.1186/1471-2431-10-98)
Supplement: Additional file 3 — Intra- and inter-observer results of subjective parameters (mean difference and limits of agreement, in parentheses). [file 1471-2431-10-98-S3.DOC]

| **Intra-observer** |  |  |  |  |
| --- | --- | --- | --- | --- |
| **Investigator** | **CP** | **MS** | **KS** |  |
| **Shape of the bony roof** | 0.98 (99.03) | 0.96 (99.03) | 0.97 (99.03) |  |
| **Position of the cartilaginous roof** | 1 (100) | 1 (100) | 1 (100) |  |
| **Inter-observer** |  |  |  |  |
| **Investigators** | **CP – MS** | **CP – KS** | **MS - KS** | **Between all investigators** |
| **Shape of the bony roof** | 0.18 (71.50) | 0.24 (71.01) | 0.17 (73.43) | 0.20 (57.97) |
| **Position of the cartilaginous roof** | 0.20 (93.72) | 0.22 (93.00) | 0.29 (93.00) | 0.24 (89.86) |
